# Range Expansion of the Jumbo Squid in the NE Pacific: δ^15^N Decrypts Multiple Origins, Migration and Habitat Use

**DOI:** 10.1371/journal.pone.0059651

**Published:** 2013-03-18

**Authors:** Rocio I. Ruiz-Cooley, Lisa T. Ballance, Matthew D. McCarthy

**Affiliations:** 1 National Research Council and Southwest Fisheries Science Center, National Marine Fisheries Service, National Oceanic and Atmospheric Administration, La Jolla, California, United States of America; 2 Southwest Fisheries Science Center, National Marine Fisheries Service, National Oceanic and Atmospheric Administration, La Jolla, California, United States of America; 3 Ocean Sciences Department, University of California Santa Cruz, Santa Cruz, California, United States of America; Institute of Marine Research, Norway

## Abstract

Coincident with climate shifts and anthropogenic perturbations, the highly voracious jumbo squid *Dosidicus gigas* reached unprecedented northern latitudes along the NE Pacific margin post 1997–98. The physical or biological drivers of this expansion, as well as its ecological consequences remain unknown. Here, novel analysis from both bulk tissues and individual amino acids (Phenylalanine; Phe and Glutamic acid; Glu) in both gladii and muscle of *D. gigas* captured in the Northern California Current System (NCCS) documents for the first time multiple geographic origins and migration. Phe δ^15^N values, a proxy for habitat baseline δ^15^N values, confirm at least three different geographic origins that were initially detected by highly variable bulk δ^15^N values in gladii for squid at small sizes (<30 cm gladii length). In contrast, bulk δ^15^N values from gladii of large squid (>60 cm) converged, indicating feeding in a common ecosystem. The strong latitudinal gradient in Phe δ^15^N values from composite muscle samples further confirmed residency at a point in time for large squid in the NCCS. These results contrast with previous ideas, and indicate that small squid are highly migratory, move into the NCCS from two or more distinct geographic origins, and use this ecosystem mainly for feeding. These results represent the first direct information on the origins, immigration and habitat use of this key “invasive” predator in the NCCS, with wide implications for understanding both the mechanisms of periodic *D. gigas* population range expansions, and effects on ecosystem trophic structure.

## Introduction

Range expansion of species has been linked to recent climate change [1] and has potentially important negative consequences for the population dynamics of native species and trophic structure and biodiversity of entire ecosystems [2]. In the NE Pacific, the most dramatic range expansion, coincident with the 1997–1998 El Niño of the Century, was documented for an *r*-selected invertebrate species, the jumbo squid (*Dosidicus gigas*) [3]. As early as 1934, *D. gigas* were occasionally observed in waters as far as 40°N [4], [5], but their occurrence far surpassed this latitude in 1997, when they were recorded in Oregon (45°N) [3], and subsequently in the Gulf of Alaska in 2005, although they remained most abundant in waters offshore of the USA and British Columbia from 2002 to 2009 [3], [6], [7]. Multiple drivers have been proposed for this dramatic expansion, including climate change, the expansion and shoaling of the oxygen minimum zone (OMZ) in the California Current [6], [7], and the depletion of tuna and bill fish populations in the eastern tropical Pacific [8]. While this species' tolerance to a wide range of temperatures and oxygen concentrations likely facilitates shifts in distribution and abundance [9], the specific environmental triggers, underlying drivers, and geographic origins of the expanding populations remain unclear.

Range expansion and high abundance of *D. gigas* has likely also altered trophic structure of the Northern California Current System (NCCS), as this species is an opportunistic voracious predator with high growth rates and energetic demands [10]. It is also the largest ommastrephid (maximum mantle length >1.5 m) with the highest potential of fecundity (>30 million eggs) of any cephalopod species [11]. In the NCCS, it consumes a wide range of prey items (n>100), including hake (*Merluccius productus*), sardine (*Sardinops sagax*) and rockfish (*Sebastes* spp.) [12], [13], all species of high economic importance. Temporal variation in recruitment and movement of this opportunistic and aggressive predator can induce considerable variation in population dynamics and life history of prey species [10]. In British Columbia, for example, regional declines in hake abundance have been attributed to *D. gigas* predation [14]. However, a major puzzle regarding *D. gigas* range expansions is that studies to date have not been able to detect long-distance migration using bulk stable isotope ratios or electronic tagging, implying that medium to large squid move only within modest latitudinal distances (∼4° of latitude) [9], [15]. The fundamental mechanism for *D. gigas* expansions is therefore unclear: it is unknown whether *D. gigas* expanded its range into the NCCS through migration, or through passive dispersal of egg masses and paralarvae.

**Figure 1:**
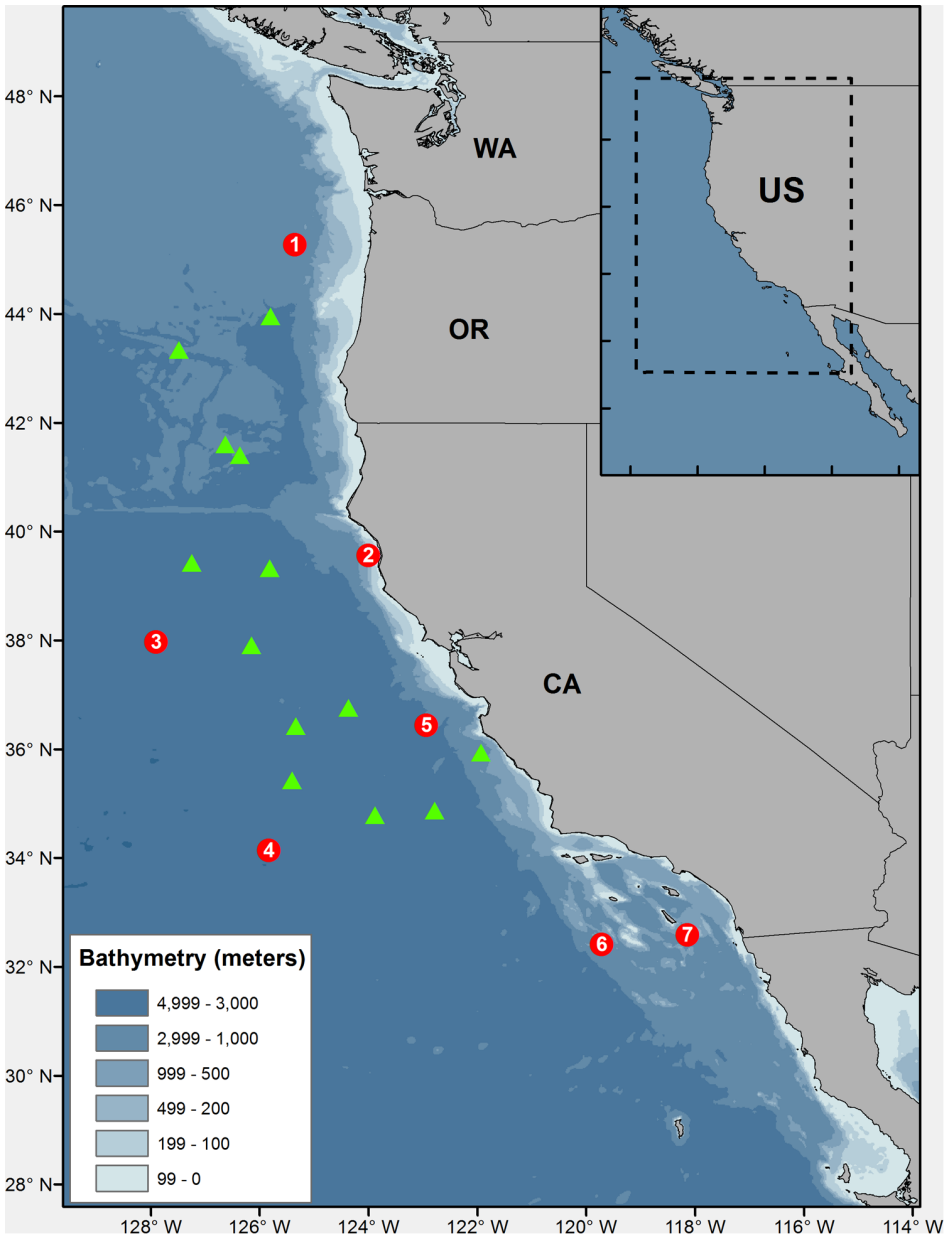
Sampling sites of *Dosidicus gigas* in 2008. Circles indicate the seven stations where squid were captured and pre-selected for stable isotope analysis. Triangles indicate other locations where squid were successfully captured.

To investigate origins, movement and habitat use of *D. gigas* inhabiting the NCCS, we analyzed stable isotope ratios of nitrogen (δ^15^N) from bulk tissue samples (gladii and muscle) and conducted compound specific isotope analysis of individual amino acids (CSIA-AA) from squid captured there (Figure 1). Natural stable isotope abundances can provide time-integrated information about the trophic position of species in a given habitat [16], [17]. In *D. gigas*, bulk isotope ratios from consecutive sections along gladii (internal shell) can also track ontogenetic shifts [15]. Isotope ratios from muscle indicate recent assimilated diet (<2 months), while consecutive sections along gladii represent a continuous dietary record throughout the period of time in which the gladius was formed [18], [19]. Because baseline δ^15^N values (i.e. values from primary producers) are heterogeneous across marine ecosystems, ocean basins and latitudes [20], movement can be detected along gladii as squid grow if *D. gigas* feed while moving between isotopically distinct habitats. However, bulk isotope analysis often cannot distinguish between shifting habitat baselines vs. trophic position.

CSIA-AA decouples these two factors by specifically identifying approximate baseline δ^15^N values for an organism, as well as shifts in trophic position [21]–[23]. Analyzing tissues from prey and taxa near the base of the food web is therefore not necessary, because trophic position can be estimated using specific source (e.g. Phenylalanine)- and trophic (Glutamic acid)- amino acid δ^15^N values coupled with equations tested under control experiments [22]. In this way, CSIA-AA has been used to document variation in specific amino acids of yellowfin tuna in the eastern tropical Pacific, indicating a relatively constant trophic position across different latitudes and longitudes, along with clear spatial differences in baseline δ^15^N values for these highly mobile predators [24], [25]. Nevertheless, CSIA-AA has so far been applied to a few taxa only, and never for cephalopods. Our study applies CSI-AA for the first time in squid, coupling its unique information with traditional bulk δ^15^N analysis to decrypt the mechanism for maintaining *D. gigas* populations in the NCCS during 2008.

## Results and Discussion

**Figure 2:**
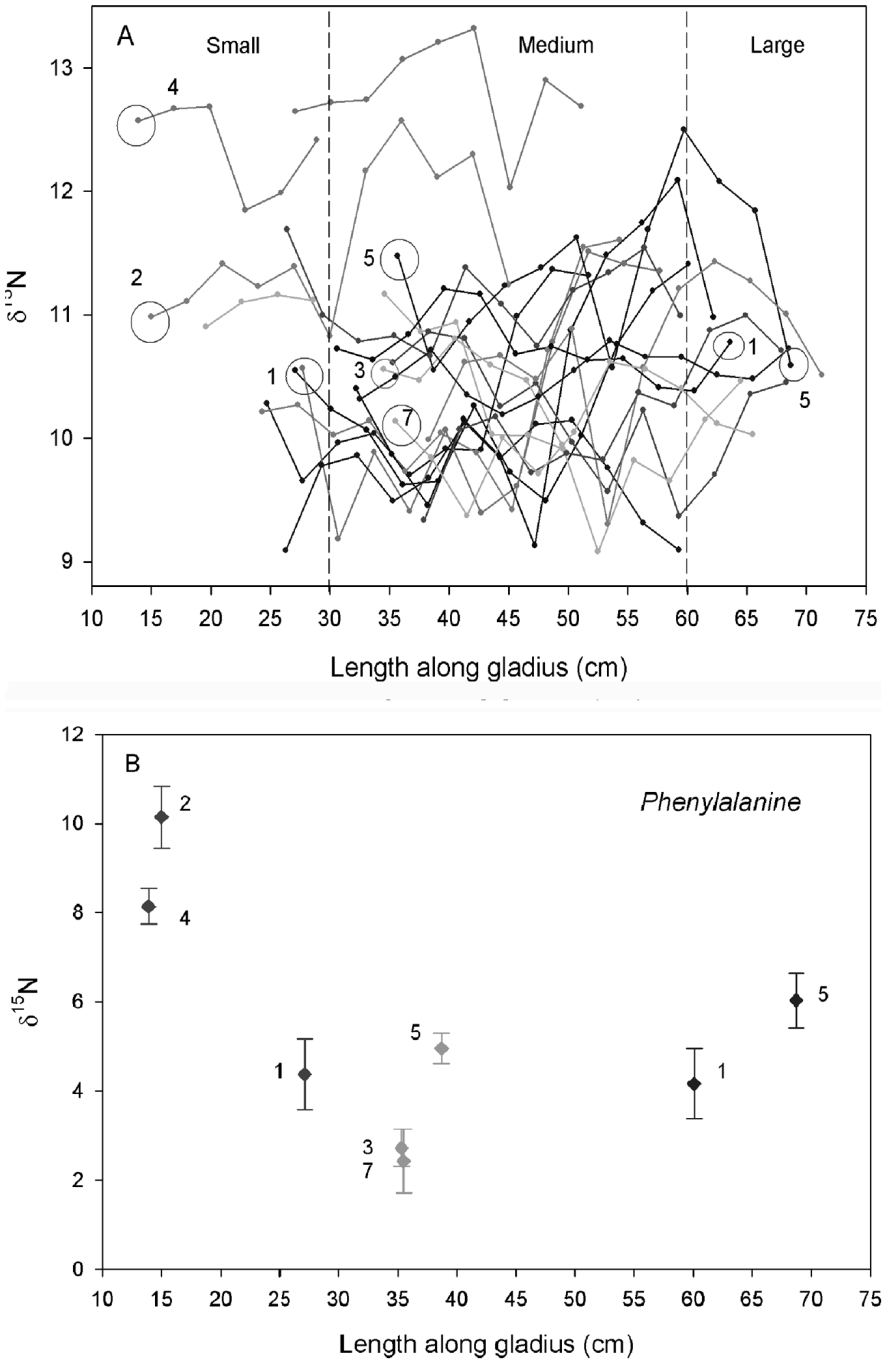
Relationship between gladii length and δ^15^N values (‰). (A) Relationship between length along gladii and bulk δ^15^N values from all proostracum sections (n = 210) of 19 squid collected at seven stations (numbers correspond to station numbers in Figure 1). Each line represents an individual squid, and dots along the line indicate δ^15^N values at each 3 cm section mean length. Open circles indicate the gladii sections of specific squid selected for further CSIA-AA. (B) Phe δ^15^N values at a given gladii length for the selected gladii sections at small, medium and large sizes.

A total of 115 squid (mean mantle length (1SD)  = 61.3 cm (8.1)) were collected from 20 stations in the NCCS, Sept – Nov, 2008 (Figure 1). Seven stations were selected *a priori* representing the maximum latitudinal and longitudinal range, for subsequent analysis of squid tissues. For these seven stations, mantle lengths ranged from 31–75 cm (mean (1SD)  = 62.2 cm (9.6)), and a total of 210 gladii sections from 19 individual squid were analyzed (δ^15^N ranged from 9‰–13.3‰). Mean bulk gladii δ^15^N values differed for each squid between the seven stations (Generalized Linear Model between stations F_6_,_12_ = 31.01, *p*<0.001, and squid nested by station F_12_,_ 190_ = 14.39, p<0.001; model R^2^ = 0.64). Comparisons of bulk δ^15^N values along the length of gladii for these 19 squid revealed generally greater variation at small sizes (<30 cm gladii length (GL), Variance  = 0.95) than larger sizes (Variance 0.8 for medium sizes: 30–60 cm GL, and 0.3 for large sizes: >60 cm GL; Figure 2A). Associated with this variation between squid at a given size was ontogenetic variation within individual squid as they grew, though the specific pattern differed between squid. Some squid had decreasing or increasing δ^15^N values as they grew, and some showed no discernible trend (Fig. 2A). These results partially differed from a previous study of *D. gigas* in the NE Pacific in which δ^15^N were also highly variable along gladii, but generally increased as a function of size [15]. These authors interpreted this variation as evidence of opportunistic predation with squid feeding at higher trophic levels as they grew in the same ecosystem.

In contrast, the δ^15^N patterns along gladii in the present study indicate migration into the NCCS from multiple origins, rather than ontogenetic shifts in diet of resident squid within the NCCS. In particular for small squid, arm size and the chitinous beak pose constraints on prey size so that small squid feed on small prey and at low trophic levels [26]. Given the same diet composition and habitat, δ^15^N from multiple small individuals should be homogenous, as opposed to the observed heterogeneous values (Figure 2A). Our further CSIA-AA analysis allows a first direct examination to distinguish long-distance migration between regions of different δ^15^N baseline values, from changes in trophic position as an explanation for change in bulk δ^15^N values. Specifically, Phenylalanine (Phe) is the most stable of the so called “source” amino acids, and provides a direct estimate for δ^15^N value at the base of food webs, while glutamic acid (Glu) is widely used as the most reliable “trophic” amino acid, central to cycling of nitrogen, and so, strongly ^15^N-enriched relative to diet [21]–[23]. Here, measured Phe δ^15^N values from a subset of gladii sections at lengths <30 cm confirm different baseline values, and therefore distinct geographic origins of small squid (Figure 2B).

Given these different Phe δ^15^N values for squid at small sizes and 35 cm GLs (Figure 2B), our CSI-AA data can suggest specific geographic regions for the origins of northern *D. gigas* population. Phe δ^15^N values represent a proxy for baseline δ^15^N values, as previously demonstrated in multiple environments [21], [23], [27]. However, while Phe δ^15^N values derived from primary production remain relatively unchanged with trophic transfer [21], [22], only approximate links to either bulk algal or local nitrate δ^15^N values can be made because (a) the exact offset between Phe and bulk algal δ^15^N values is not yet precisely known [22], and (b) if inorganic nitrogen is not completely utilized at a given location or time period where squid fed, additional isotopic fractionation between local nitrate and algal δ^15^N values would be expected [28].

Considering the known geographic patterns in baseline δ^15^N value variation in the N Pacific [29]–[31], one geographic origin likely includes waters of northern Baja California, Mexico, since Phe δ^15^N values (4–8‰) correspond to δ^15^N-NO_3_^−^ values from 100–200m depths between 28°N and 35°N (5–8.5‰) [29]. Direct observations support this, as small- to medium-sized *D. gigas* (21–35.9 cm MLs) have been collected offshore of N Baja California, Mexico (30 July, 2006 at 29°N and 116°W; R. I. Ruiz-Cooley, squid collection). A second possible source region, corresponding to the highest Phe δ^15^N values observed in our squid (10‰), includes regions of active denitrification such as waters of the southern Baja California Peninsula (e.g., 22–23°N; δ^15^N-NO_3_^−^  = 9-13‰) [29]. Lastly, a third possible source region corresponds to the lowest Phe δ^15^N values (2–3‰) measured (see squid at 35 cm GLs; Figure 2B). These low values are not consistent with δ^15^N-NO_3_^−^ values in the CCS, British Columbia or eastern tropical Pacific [30], While it cannot be ruled out that these squid values may reflect regions of replete surface nitrate (where strong isotope fractionation favors the lighter isotope [28]), such depleted δ^15^N values likely indicate a source region where N fixation influences primary production δ^15^N values. Both ocean temperatures and the influence of N fixation increase rapidly moving offshore from the main CCS into the edge of the more oligotrophic gyre [32]. Therefore these values could be consistent with a source region in much warmer offshore waters to the west, perhaps near the boundary of the north Pacific Gyre (NPSG) system.

**Figure 3:**
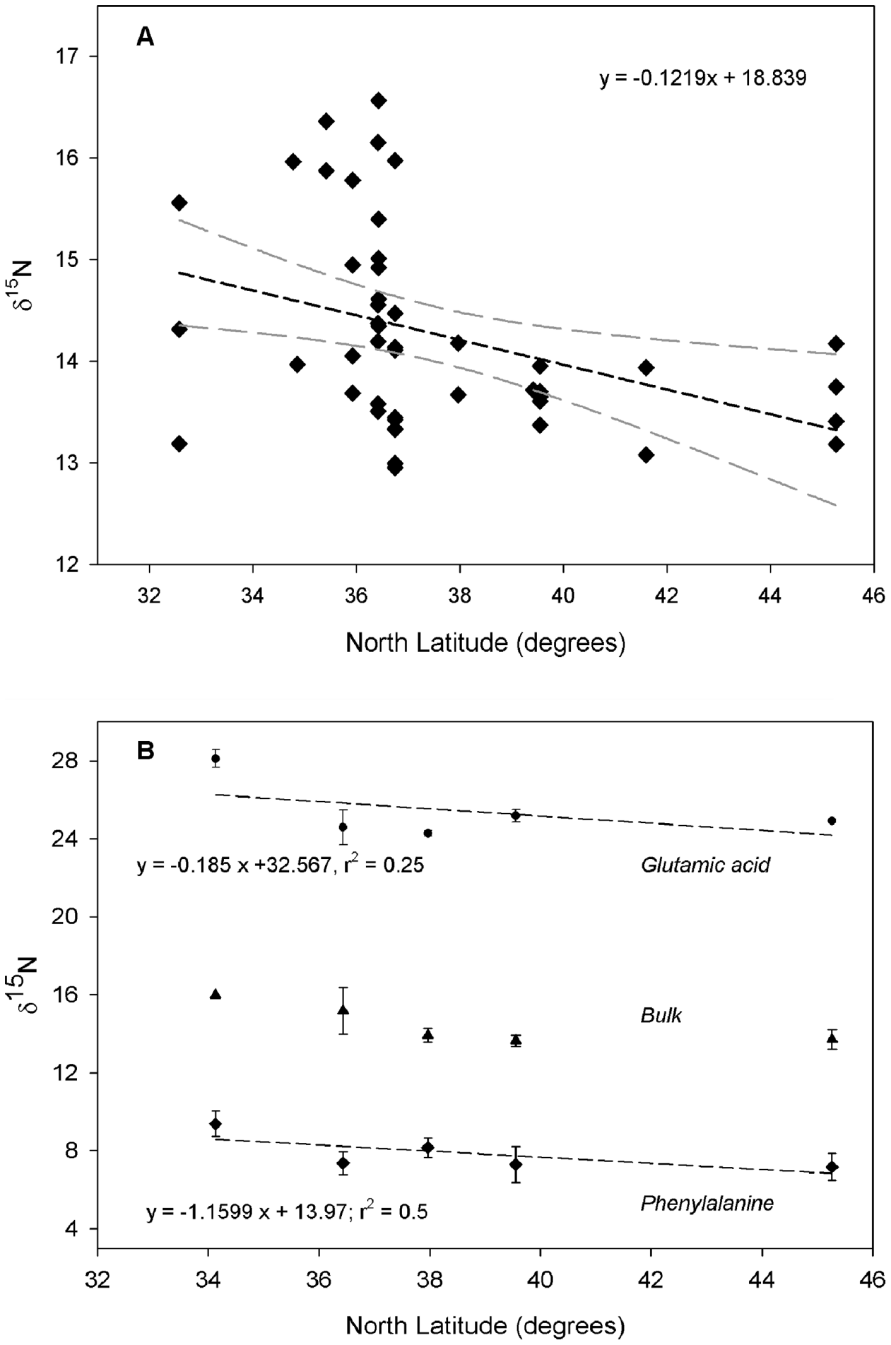
*Dosidicus gigas* latitudinal variation in δ^15^N values (‰). (A) Latitudinal variation in bulk δ^15^N values (n = 45) from muscle and (B) amino acid compound specific data from composite muscle samples. Sample size for each composite sample in a given stations is: n = 3 squid for stations 1, 2 and 5; n = 2 squid for station 3; n = 1 squid for station 4. Mean and one standard deviation are shown for Phenylalanine (the main source amino acid;⧫), Glutamic acid (the main trophic amino acid; ▪), and bulk (▴) δ^15^N. Dashed lines indicate linear regressions: (A) δ^15^N_bulk_, r^2^ = 0.14, p<0.01; (B) δ^15^N_Glutamic acid_, p = 0.4 and δ^15^N_Phenylalanine_, p = 0.1.

In contrast to the high variation in δ^15^N for small squid, bulk δ^15^N and Phe δ^15^N values for squid larger than 60 cm exhibited low variation (Figure 2A, B), indicating a common feeding area for large animals. Further, Phe and Glu δ^15^N values in composite muscle tissue samples exhibited a latitudinal gradient, with progressively decreasing values as latitude increased from 33°N to 46°N (Figure 3). Following the approach of Popp et al [24], the observation that change in Glu δ^15^N parallels that in Phe δ^15^N indicates that the decreasing bulk δ^15^N observed with increasing latitude is due to spatial change in baseline isotopic values, and not to shifts in trophic position. Furthermore, Glu-Phe Δ^15^N values, representing a direct proxy for relative trophic position, remained constant as a function of latitude (Glu-Phe Δ^15^N ranged from 16.1 to 18.7‰; Figure 3B). The decreasing trend in both bulk and CSIA-AA is strongly consistent with the expected latitudinal gradient in baseline δ^15^N values in the NCCS, as documented by multiple sample types, including upper water column of NO_3_^−^, organic matter from both sediment traps and sediments collected from southern California to Oregon [30], [31], and also from *D. gigas* muscle collected in 2006 [33]. This baseline δ^15^N attenuation with latitude is fundamentally driven by California Undercurrent northward transport of elevated δ^15^NO_3_^−^ waters originating in the strongly denitrifying eastern tropical Pacific [30], [31], [34], while lower salinity and higher dissolved O_2_ surface waters of the California Current are transported equatorward [35]. The fact that a δ^15^N latitudinal gradient for three muscle tissue isotopic measurements (bulk, Phe and Glu) all match the expected gradient in baseline δ^15^N values for the NCCS, indicates residence of large squid at specific latitudes for time periods long enough to integrate local marine biochemical processes.

Such “regional” residence for this highly mobile predator might seem unexpected based on prior tagging experiments. Squid larger than 70 cm ML have been shown to move within a particular ecosystem (i.e., only within the central Gulf of California) over distances as great as180 km in one week [9], and mainly between inshore-offshore waters in the NCCS [36]. However, maturity data and size frequencies of 870 jumbo squid support the idea that jumbo squid move seasonally and large mature females leave the NCCS abruptly in the fall to spawn in Mexican waters [13]. If large squid do move seasonally, why would Phe δ^15^N in muscle track the expected latitudinal δ^15^N baseline gradient? A likely explanation unifying all these observations is that the δ^15^N isotopic equilibration rates for squid muscle may be faster than previous estimates of 2 months [19]. This rate was calculated based on a simple dilution model and available growth rates, from the population in the Gulf of California. Since only the large morph is found in the NCCS [11], our data suggest that new equilibration rates are needed once growth rates are available for this system. Furthermore, once females leave the NCC, they possibly remain in spawning areas until they release all their eggs and die. *D. gigas* are intermittent, multiple spawners and can release 5–30 million eggs they produce over a lifetime (oviducts can hold approximately 1.2 million eggs at any one time) within an approximate period of 3 to 4 months) [37]. The energy cost for spawning is expected to be high, and no long-distance migrations would be expected during its spawning period.

This study demonstrates how δ^15^N from bulk tissues coupled with CSIA-AA of a highly dynamic *r*-selected species can reveal distinct geographic origins and ontogenetic changes in habitat use and residence patterns. Our data indicate that *D. gigas* inhabiting waters from 32° to 45°N latitude (Figure 1) in fact emigrated from multiple regions prior to reaching their largest size. Once in the NCCS, these large squid remained and fed in specific regions, at least long enough to integrate baseline isotopic values. Fisheries data indicate that other species of ommastrephids (e.g., *Illex* sp.) may be capable of migrating over long distances for spawning, reproduction and feeding [38]. Our study provides the first direct evidence that small ommastrephids do migrate, likely over long-distances and between ecosystems that are biochemically distinctive. For *D. gigas*, there are no direct observations of mass migrations, but multiple strandings recorded along the coasts of California, Oregon, and Canada [39] are consistent with the idea that such migrations do occur.

Together, these results have important implications for understanding the influence of climate change on range expansions. The appearance and high abundance of *D. gigas* in CA occurred post-1997 and 2002, years of warm SST in the eastern tropical Pacific [8]. To date, paralarvae of jumbo squid have not been found in waters north of the U.S.-Mexico border [40], and laboratory experiments have confirmed that eggs can successfully develop only at water temperatures between 15°C and 25°C [41]. Because larvae and small sized-squid have a lower thermal tolerance than large squid [42], we hypothesize that wider distribution for spawning eggs and higher success in egg development during anomalously high temperature events (e.g., El Niño events), coupled with migration of smaller squid, promote range expansions of *D. gigas*. Under this scenario, small-sized squid migrate toward the NCCS to feed actively, as shown by stomach content analysis [13], grow until they mature, and likely return to warmer waters to spawn multiple times in regions that include coastal and offshore Mexican waters along the Baja California peninsula, and possibly the edge of NPSG. Finally, because *D. gigas* is a short lived species (∼1.5 years), the fact that our data show multiple origins for individuals collected from the NCCS in a single year (2008), coupled with observations that the species remained abundant in this ecosystem for multiple years (2002–2009) [8], [13], indicates that multiple immigrations over time were required to maintain the NCCS population. This hypothesis conflicts with a previous one which proposed a sustained northern population with a lack of multiple invasions [8]. Both expansion of OMZ areas and increased fisheries pressure on top predators have been proposed as factors that triggered *D. gigas* range expansions. However, both these factors have continued to accelerate, while the population of *D. gigas* in the NE Pacific has contracted since 2009 [43] These observations, together with the data documented here, strongly support the idea that El Niño years in 1997 and 2002 were most responsible for triggering the multiple migrations that lead to the recent and long-lasting range expansion of *D. gigas*.

## Materials and Methods

Squid were collected by jigging under a bright light during hour-long nightly oceanographic stations, each of which began one hour after sunset. Squid sampling was part of a larger cetacean and ecosystem assessment survey of the NCCS conducted by NOAA [44]. Within an hour of capture, squid were measured and frozen at −20°C. Additional processing was conducted post survey. For each, the gladius was extracted, measured and the proostracum (that portion of the gladius formed by the ostracum [45]) was cut transversely every 3 cm sections and prepared for stable isotope analysis (SIA) following Ruiz-Cooley et al. [15]. Details of bulk δ^15^N analysis (‰) are provided elsewhere. A GLM with nested factors (each squid nested by station) was used to compare the effect of sampling location for a given squid δ^15^N values on gladii. In addition, squid were classified into three size classes: small (<30 cm GL), medium 30–60 cm GLs) and large (>60 cm GLs), to investigate ontogenetic variation. Muscle tissue from the mantle (of 19 squid from the 7 geographic extreme stations plus an additional 28 squid randomly selected from all remaining stations) was prepared for SIA following Ruiz-Cooley and Gerrodette [33]. Linear regression was performed to examine the relationship between latitude and δ^15^N values.

For CSIA-AA, 3.5–4 mg of a subset of gladii sections (n = 8) at small, medium and large squid lengths (see Figure 2) and five muscle composite samples (each composed of different squid randomly selected for a given station; see caption Figure 3B) were analyzed following previously published protocols [46]. Briefly, individual AA isotopic analyses were made on acid hydrolysates (6 N HCl, 100 ml, 20 h) of homogenized material, following the formation of isopropyl-TFA derivatives. Derivatives were analyzed on a Thermo Trace Ultra GC, fitted with a Agilent DB-5 column (50 m×0.32 mm i.d. ×0.52 um film thickness), in line with an oxidation furnace and reduction furnace, and linked to a Finnigan Delta^Plus^ XP mass spectrometer. All samples were derivatized using an accompanying AA external standard for which authentic δ^15^N values of each AA were determined offline, to monitor the accuracy of the instrument; an internal standard (Nor-Leu) was also added to each sample hydrolysate. Samples were injected in quadruplicate, bracketed by standards, and sample δ^15^N values were corrected based on a running average of known standard values through a run. Reproducibility for individual AA values was typically better than 1‰.
